# Removal of 1,4-Naphthoquinone by Birnessite-Catalyzed Oxidation: Effect of Phenolic Mediators and the Reaction Pathway

**DOI:** 10.3390/ijerph17134853

**Published:** 2020-07-06

**Authors:** Han-Saem Lee, Jin Hur, Doo-Hee Lee, Mark A. Schlautman, Hyun-Sang Shin

**Affiliations:** 1 Department of Environment Energy Engineering, Seoul National University of Science & Technology, Seoul 01811, Korea; hansun213@seoultech.ac.kr; 2 Department of Environment & Energy, Sejong University, Seoul 05006, Korea; jinhur@sejong.ac.kr; 3 Mass Spectrometer Laboratory, National Instrumentation Center for Environmental Management, Seoul 08826, Korea; dohe80@snu.ac.kr; 4 Department of Environmental Engineering and Earth Science, Clemson University, Clemson, SA 29634, USA; mschlau@clemson.edu

**Keywords:** 1,4-naphthoquinone, birnessite, phenolic mediator, cross-coupling, kinetics, pathway

## Abstract

This study investigated the birnessite (*δ*-MnO_2_) catalyzed oxidative removal of 1,4-naphthoquinone (1,4-NPQ) in the presence of phenolic mediators; specifically, the kinetics of 1,4-NPQ removal under various conditions was examined, and the reaction pathway of 1,4-NPQ was verified by liquid chromatography–tandem mass spectrometry (LC–MS/MS). The removal rate of 1,4-NPQ by birnessite-catalyzed oxidation (pH = 5) was faster in the presence of phenolic mediators with electron-donating substituents (pseudo-first-order initial stage rate constant (*k*_1_) = 0.380–0.733 h^−1^) than with electron-withdrawing substituents (*k*_1_ = 0.071–0.244 h^−1^), and the effect on the substituents showed a positive correlation with the Hammett constant (Σσ) (*r*^2^ = 0.85, *p* < 0.001). The rate constants obtained using variable birnessite loadings (0.1–1.0 g L^−1^), catechol concentrations (0.1–1.0 mM), and reaction sequences indicate that phenolic mediators are the major limiting factor for the cross-coupling reaction of 1,4-NPQ in the initial reaction stages, whereas the birnessite-catalyzed surface reaction acts as the major limiting factor in the later reaction stages. This was explained by the operation of two different reaction mechanisms and reaction products identified by LC-MS/MS.

## 1. Introduction

Polycyclic aromatic hydrocarbons (PAHs) are contaminants that are commonly found in water, soil, and airborne particulates and are converted into various toxic derivatives upon degradation [1,2,3]. Natural weathering, photolysis, and biochemical transformations afford oxygenated PAHs (OPAHs) [4,5,6,7]. On the basis of detailed soil pollutant analyses, Sehlin [8] concluded that the natural weathering of contaminated soil can result in the OPAH and PAH contents remaining at high levels for long periods; it was also demonstrated that the incomplete biochemical degradation and conversion of PAHs in contaminated soil or water leads to OPAH generation and accumulation. Notably, Ferrarese et al. [9] showed that, although PAH removal efficiencies exceeding 90% can be achieved using the Fenton reaction, persulfate, H_2_O_2_/permanganic acid, and other chemical oxidation methods, the corresponding total organic carbon removal is much lower (50–80%), which suggests that residual organic carbon is mainly present as partially oxidized PAHs, i.e., OPAHs. The importance of OPAHs has been increasingly recognized [3,5]. For example, Woo et al. [10] found alcohol, ketone, quinone, aldehydes-based OPAH compounds during the photocatalytic oxidation of PAH, and the maximum yield of intermediates can be up to 40% in the case of the degradation of anthracene. Additionally, Kuppusamy et al. [11] reported in a review paper on remediation techniques, including thermal, chemical, and biological treatments that have been widely applied to PAH-contaminated soils. The complete restoration of the contaminated soil to its natural state is impractical and suggested the risk-based remediation approach toward reducing ecological toxicity.

The term “OPAH” encompasses a broad variety of oxygen-containing PAH derivatives (e.g., ketones, quinones, aldehydes, carboxylic acids, and phenols) that are frequently more toxic than the original PAHs [4,12]. Moreover, the increased solubility and bioavailability of OPAHs compared with those of PAHs facilitate the diffusion of the former into the groundwater and the surrounding environment, which results in an increased environmental impact [8,12]. In particular, ketone- and quinone-based OPAHs exhibit higher toxicity and persistence than the corresponding phenols and carboxylic acids and are, thus, more harmful [13,14]. 1,4-naphthoquinone (1,4-NPQ), a representative quinone-based OPAH (e.g., quinoid PAH) commonly produced by the biochemical degradation of naphthalene- and phenol-containing PAHs [15,16], can bind to macromolecules such as DNA and proteins, making it a potential tumorigenic agent [13]. However, most studies of the decomposition and removal of PAHs have focused on the removal of the original pollutants (i.e., PAHs), and the subsequent treatment and effects of the oxidative transformation of PAH products remains underexplored.

Birnessite (*δ*-MnO_2_) is a naturally occurring manganese oxide that has a relatively high specific surface area (approximately 30–300 m^2^ g^−1^) and an amorphous structure; interestingly, it promotes the formation of naturally occurring polymers (e.g., humic substances) from phenolic compounds via non-biological oxidative coupling [17,18,19]. Recent investigations have shown that organic contaminants that are intrinsically non-reactive or only weakly reactive toward birnessite (e.g., sulfonamide, prions, and endocrine disrupting chemicals) can be removed by birnessite-catalyzed cross-coupling in the presence of hydroxyl-containing mediators [20,21]. For example, Song et al. [20] reported that the MnO_2_-mediated transformation of sulfonamides occurs in the presence of syringaldehyde, while MnO_2_ alone has only a limited effect on the transformation of these compounds. Wang et al. [21] also showed that the transformation of phenolic endocrine disrupting chemicals was enhanced with the addition of simple phenol as a cosubstrate. The above findings imply that birnessite-catalyzed cross-coupling can be widely applied for the removal of various recalcitrant aromatic compounds, including pesticides and antibiotics. Therefore, studies on the removal of quinone OPAHs by birnessite are expected to have important implications not only for the removal of these contaminants from water and soil but also for the development of better remediation methods for PAH-contaminated soils and water in conjunction with existing biological and chemical treatment techniques.

In this study, we investigated the oxidative removal of 1,4-NPQ by birnessite in the presence of various phenolic mediators and determined the effects of the mediator structure on the removal efficiency. In addition, we analyzed the birnessite-mediated reaction products of 1,4-NPQ in the presence of a phenolic mediator using liquid chromatography-tandem mass spectrometry (LC-MS/MS) and studied the effects of birnessite/phenolic mediator loading and the sequence of reactions between 1,4-NPQ, birnessite, and catechol (CAT) on the time-dependent efficiency of 1,4-NPQ removal. The main purposes of this study were to provide that the application of the birnessite-catalyzed cross-coupling reaction is expandable to the removal of quinoid PAH compounds and obtain insights into the structural effects of the phenolic mediator and, the mechanism and kinetics of the removal process.

## 2. Materials and Methods

### 2.1. 1,4-NPQ and Birnessite

1,4-NPQ and phenolic compounds (all >99%) were obtained from Sigma-Aldrich and used without further purification (Table S1). The eleven phenolic compounds used in this study are the major structural units of humic substances and are frequently used as model humic constituents [22,23]. A 0.35-mM stock solution was prepared by dissolving 1,4-NPQ into deionized water and stirring the obtained solution for 24 h under N_2_ followed by filtration through a disk-type polytetrafluoroethylene syringe filter (0.45 μm, Toyo Roshi Kaisha Ltd., Japan). Birnessite was prepared by boiling potassium permanganate in hydrochloric acid as described by McKenzie [24]. The resulting oxide particles were filtered and washed repeatedly with deionized water to ensure the removal of excess reagents and then freeze dried.

### 2.2. 1,4-NPQ Removal Experiments

The reaction of 1,4-NPQ with birnessite in the presence of the reactive mediators was investigated by performing batch tests in 20-mL serum bottles. The reaction solution was prepared by mixing the 1,4-NPQ standard solution (0.14 mM, pH = 5) with a solution of the phenolic compound (0.6 mM, pH = 5) at a 1:1 (*v*/*v*) ratio, followed by the immediate injection of birnessite to achieve a loading of 1.0 g L^−1^. Here, pH = 5 was selected as a weakly acidic region that can exhibit high reactivity considering the p*k*a value (4.3–9.9) of the phenolic reaction mediators and the point of zero charge (PZC, 2.4–2.7) of birnessite based on the previous research results [15,17]. For comparison, a reactive mediator-free control sample solution was prepared under the same conditions (0.07 mM 1,4-NPQ, 1.0 g L^−1^ *δ*-MnO_2_, pH = 5). The pH of the reaction solution was adjusted to 5 using HCl and NaOH. At this pH, the reactivity of birnessite is the most favorable, and this pH also mimics that of natural water bodies (typically 5–8) [15]. The reaction vessel was completely sealed with a Teflon diaphragm and Al cap, and Al foil was used to block light. The reaction mixture was stirred at 30 rpm using a rotary stirrer (AG, FINEPCR^®^, Gunpo, Korea), and 500-μL aliquots were collected at appropriate time intervals over 24 h and filtered through a 0.45-μm syringe filter. The concentrations of the dissolved and adsorbed Mn ions in the reaction slurry (after a reaction time of 24 h) were analyzed by centrifugation and sediment washing with 2 M KCl according to the method of Chang Chien et al. [25]. The effects of the birnessite (0.1–1.0 g L^−1^) and CAT (0.1–1.0 mM) loadings were determined by varying each of these variables while keeping the other parameters constant (Figure S1). For the CAT loading experiments, birnessite was injected at a concentration of 0.5 g L^−1^. The effect of the reaction sequence was evaluated using two different batches. In the case of batch 1 (B1), the reaction solution (pH = 5.0) was prepared by adding birnessite to a mixed solution of 1,4-NPQ (0.07 mM) and CAT (0.5 mM), whereas, for batch 2 (B2), a mixed solution of birnessite and CAT (0.5 mM) was incubated for 30 min and then treated with 1,4-NPQ (0.07 mM).

### 2.3. Analytical Measurements

FT/IR spectrum (Bomen MB154) of the birnessite showed characteristic peaks at 930, 1630 and 3450 cm^−^^1^ [26]. X-ray diffractometry (XRD, D/Max-III C, Rigaku Denki Co., Tokyo, Japan) analysis showed that the prepared manganese oxide contained birnessite (δ-MnO_2_) as the major crystalline phase, having reflections at 2 θ values of 12.2°, 24.3°, 36.7°, and 66.4° (Figure S2) [27]. The specific surface area of the product was measured using the Brunauer-Emmett-Teller N_2_ gas adsorption method and found to be 47.29 m^2^ g^−1^. The crystal phase of birnessite (measured using JSM-6700F, JEOL Ltd., Tokyo, Japan.) showed an amorphous cloud-like shape formed by the aggregation of small particles and was less than 4.0 μm (Figure S3). The concentrations of 1,4-NPQ and the phenolic mediators for the analysis of reaction kinetics were determined by high-performance liquid chromatography (HPLC; ACME 9000, Younglin Ltd., Anyang, Korea) using a reverse-phase column (C18, 4.6 × 150 mm, 5-μm particle size, Atlantis^®^, Waters, Newcastle, DE, USA). The flow rate of the mobile phase (50 vol % aqueous acetonitrile) was 1.0 mL min^−1^, and 50-μL samples were injected and analyzed (UV detection at 254 nm). The spectrometric analysis of the reaction products was performed using the LTQ XL linear ion trap tandem mass spectrometer (LC-MS/MS, Thermo Scientific, Waltham, MA, USA) interfaced with the Ultimate 3000 HPLC system (Thermo Scientific, Waltham, MA, USA) equipped with an Agilent Eclipse C18 column (2.1 × 150 mm, 3.5-μm particle size). The mobile phase was water (A) and 100% acetonitrile (B) in gradient mode (A:B = 95:5 (1’) to 5:95 (15’)) at a flow rate of 0.3 mL min^−1^. Samples were ionized by electrospray ionization in negative modes. The operating parameters were as follows: ion source voltage, 3.5 kV; capillary temperature, 320 °C; capillary voltage, 10 V; tube lens voltage, 55 V; normalized collision energy, 35 eV; and scan range, *m/z* 50–2000. The Mn concentration was measured by atomic absorption spectroscopy (AAS, UK/Solaar AAS-M6, Thermo Scientific, Waltham, MA, USA). The Hammett constants for the phenolic mediators used in this study were derived using the Hammett equation (Equation (1)).

(1) $$log\left(k/k_{h}\right)=\rho \Sigma \sigma ,$$

In Equation (1), *k* and *k_h_* are the reaction rate constants of substituted and unsubstituted phenolic compounds, respectively, *ρ* is the susceptibility factor, and Σσ is the sum of the Hammett constants [28,29].

## 3. Results and Discussion

### 3.1. Effect of Phenolic Mediators on the Efficiency of 1,4-NPQ Removal by Birnessite

Table 1 lists the 1,4-NPQ removal efficiencies of birnessite (as averages of three repeated measurements) achieved in the presence of the eleven phenolic mediators after 24 h incubation. Notably, a removal efficiency of 1.7% was observed in the absence of birnessite and reactive mediators, which was ascribed to the adsorption of 1,4-NPQ onto the sample container walls and, possibly, volatilization, whereas a removal efficiency of 9.8% was observed in the presence of birnessite only. Thus, the ability of birnessite to remove 1,4-NPQ by itself is quite low, as reported previously [15]. However, removal efficiencies of 77.6–100% for 1,4-NPQ were obtained for birnessite in the presence of phenolic mediators, and particularly high values (>99.8%) were achieved for species containing two phenolic groups (i.e., CAT, hydroquinone (HQ), and resorcinol (RES)) or methoxy groups (i.e., 4-methoxyphenol (4-MeP) and 2,6-dimethoxyphenol (2,6-DiMeP) after 24 h. The removal of 1,4-NPQ by birnessite-mediated oxidative reactions was confirmed by comparing the concentrations of Mn ions in the 1,4-NPQ and birnessite reaction samples in the presence and absence of CAT. In the absence of 1,4-NPQ or CAT, no dissolved Mn was detected in the birnessite suspension at pH = 5, whereas appreciable amounts of dissolved and adsorbed Mn (total 1.90 μmol) were detected in the 1,4-NPQ-birnessite reaction system (Table S2), indicating the reduction and subsequent dissolution of Mn from birnessite during the catalytic oxidation of 1,4-NPQ. Similarly, Chang Chien et al. [25] reported that the change in the oxidation number of Mn in birnessite and the subsequent release of dissolved Mn corresponds to the reduction of birnessite in the catalytic oxidative degradation of phenolic compounds, and the higher catalytic polymerization or mineralization of the converted phenolic compounds leads to a higher ratio in the amount of phenolic compound oxidized compared with that of Mn released in *δ*-MnO_2_. This interpretation is confirmed by the significantly increased concentration of reduced Mn (total 155.5 μmol) produced from birnessite and based on the removal of the majority of 1,4-NPQ in the presence of CAT as a phenolic mediator (Table S2). On the other hand, the relative concentration (*C*/*C*_0_) of 1,4-NPQ in the presence of individual phenolic compounds showed that the initial rapid decrease in concentration subsequently slowed (Figure S4), which has been ascribed to the gradual retardation of 1,4-NPQ cross-coupling in the presence of phenolic mediators with increasing reaction time [23,30].

**Figure 1:**
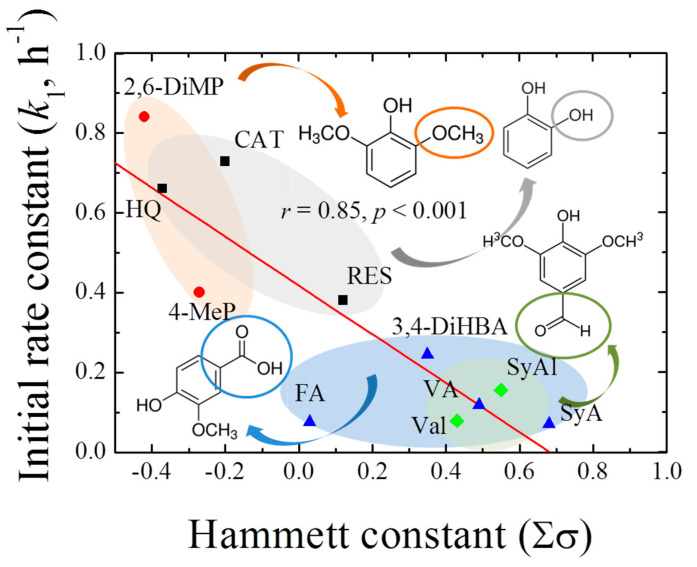
Correlation between the pseudo-first-order rate constant (*k*_1_, h^−1^) for 1,4-NPQ removal by birnessite-catalyzed oxidation and the Hammett constants (Σσ) of the phenolic mediators.

Based on the above results, the rate constants in the initial stages of 1,4-NPQ removal (*k*_1_, h^−1^) in the presence of individual phenolic mediators were obtained by fitting the experimental data to the pseudo-first-order kinetic model (Table 1). The initial reaction period for kinetic analysis was determined based on the coefficients of determination (*r*^2^ ≥ 0.98) for the fitting of the first-order model to the data. The reaction time and percent initial removal efficiency were different for the different phenolic mediators. For the diphenolic and methoxy phenolic compounds, the removal rate ranged from 46.7 to 82.3% for up to 1.5 h, whereas for acidic- and aldehyde-phenols, a relatively low removal rate in the range 15.5–38.7% (up to 2 h) was observed. The thus-determined *k*_1_ values ranged from 0.071 to 0.733 h^−1^, depending on the mediator structure. Among the diphenolic compounds, the highest *k*_1_ was observed for CAT (0.733 h^−1^), followed by those of HQ (0.611 h^−1^) and RES (0.380 h^−1^). Therefore, CAT and HQ, which have hydroxyl groups at the *o*- and *p*-positions of the benzene ring, were more effective mediators than RES, which has a *meta*-hydroxyl group. Similarly, Shindo [31] reported that the efficiencies of oxidative diphenolic compound removal by birnessite were in the order CAT > HQ >> RES, and Kennedy et al. [32] reported that, in the electrochemical polymerization of phenols and anilines, the efficiency of the oxidative polymerization of RES was relatively low. Based on these reports, the phenolic compounds exhibiting high reactivity toward birnessite were expected to undergo highly efficient cross-coupling reactions with 1,4-NPQ, and CAT was identified as the most reactive mediator for the oxidative removal of 1,4-NPQ among the diphenolic compounds. Moreover, high *k*_1_ values of 0.657 and 0.400 h^−1^ were observed for the methoxy-containing 2,6-DiMeP and 4-MeP mediators, respectively, whereas relatively low rate constants of 0.071–0.244 h^−1^ were observed for mediators containing carboxyl and aldehyde groups. The results were further confirmed by the plot of the pseudo-first-order initial rate constants against the Hammett constants (Σσ) for each phenolic mediator (Figure 1), which showed a good correlation (*r*^2^ = 0.85, *p* < 0.001), having a slope of −0.61 (i.e., ρ). The negative sign of the susceptibility factor (ρ) indicates that the reaction is favored by increasing electron density on the aromatic ring [33]. Similarly, Choi et al. [34] reported that the removal of 1-indanone, a ketonic OPAH compound, by birnessite also showed higher reaction efficiency in the presence of CAT. However, the removal rate (*k*_1_) of 1-indanone reported is 0.041 h^−1^ (*r*^2^ = 0.86), which is about 18-times lower than that of 1,4-NPQ (0.733, *r*^2^ = 0.98). These results indicate that phenolic compounds containing electron-donating groups were more effective mediators for the birnessite-catalyzed cross-coupling reaction of 1,4-NPQ than those containing electron-withdrawing groups. On the other hand, the rate constants in the later stages of the reaction (*k*_2_, h^−1^, 2–24 h) increased with increasing *k*_1_, and there was a highly positive correlation between the two rate constants (*r*^2^ = 0.97, *p* < 0.001) (Figure S5), indicating that, regardless of the structure of the phenolic mediators, the initial rate of reaction for 1,4-NPQ removal has a direct effect on the subsequent reaction rate of 1,4-NPQ removal in the birnessite reaction system, and the *k*_2_/*k*_1_ ratio under these experimental conditions was 0.67.

### 3.2. Cross-Coupling Reaction of 1,4-NPQ by Birnessite in the Presence of CAT

To confirm the 1,4-NPQ removal process by birnessite in the presence of a phenolic mediator, the reaction products were analyzed. CAT was selected as the reactive mediator in this study because it yields the highest 1,4-NPQ removal efficiency. The intensity of the 1,4-NPQ peak (retention time (RT) = 3.62 min) decreased by 86.9% after 2-h incubation, and reaction products were observed as broad peaks with RT ≈ 1.1 min (Figure S6a). Further incubation (2–24 h) resulted in a continuous decrease in the intensity of the 1,4-NPQ peak and a concomitant increase in the intensities of the reaction product peaks, and most of the 1,4-NPQ (>99.9%) was removed after 24 h. Under the same conditions, the CAT peaks (RT = 1.83 min, Figure S6b) rapidly disappeared within 20 min, and the oxidation product peaks that appeared concomitantly (RT ≈ 1.1 min) were positioned similarly to the product peaks for 1,4-NPQ oxidation, albeit with much lower intensities. Considering that the efficiency of 1,4-NPQ removal by birnessite-mediated oxidative- transformation in the absence of phenolic mediators was around 9.8% (Table 1), it is suggested that the oxidation of CAT significantly enhanced the removal of 1,4-NPQ via birnessite-catalyzed cross-coupling reactions [23,35]. This result also proves that the 1,4-NPQ generated as an intermediate in the oxidative removal of 1-naphthol by birnessite can be further removed by the birnessite-catalyzed cross-coupling reaction with phenolic mediators [15].

**Figure 2:**
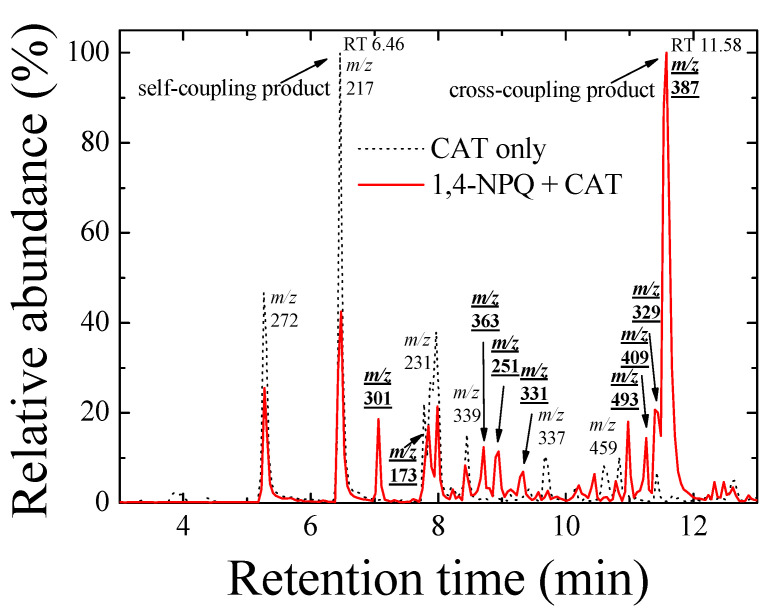
Total ion chromatogram (TIC) spectra of the reaction products of 1,4-NPQ in the presence of catechol (CAT) (solid line) and reaction products of CAT only (dotted line) by birnessite-catalyzed oxidation at a reaction time of 10 min in negative ionization mode. The numbers indicate the main mass signals, and the bold and underlined numbers indicate the mass signals for the reaction products of 1,4-NPQ in the presence of CAT. Experimental conditions: 0.07 mM 1,4-NPQ, 0.3 mM phenolic mediator, 1.0 g L^−1^ *δ*-MnO_2_, 20 °C, and pH = 5 in the dark.

**Figure 3:**
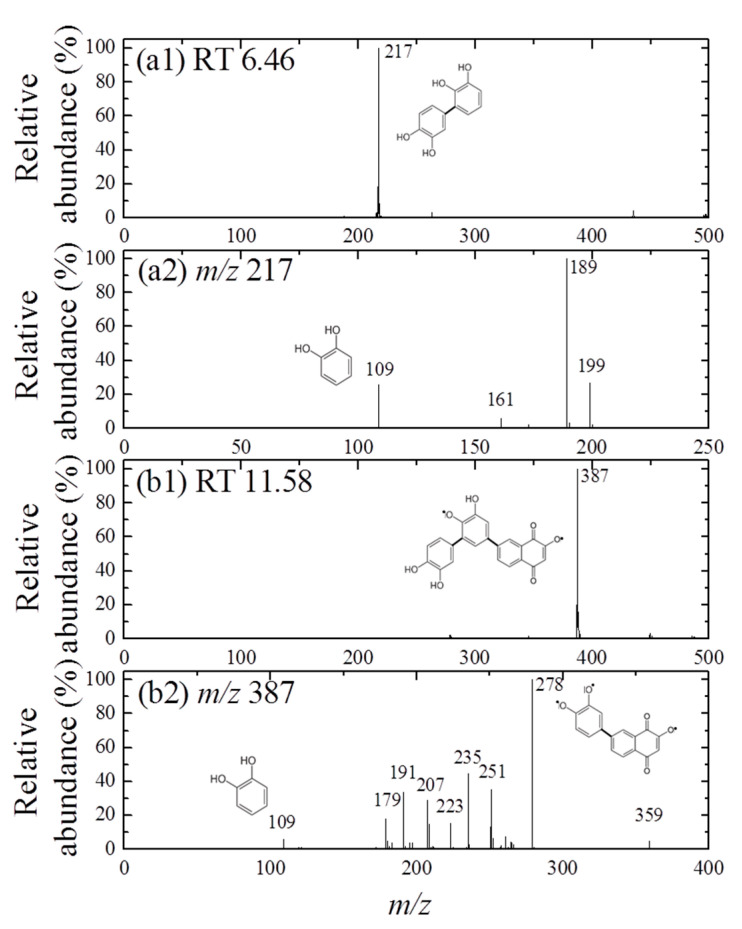
MS spectra (**a1**,**b1**) and MS/MS spectra (**a2**,**b2**) obtained for the mass peaks at (**a**) m/z 217(CAT only, RT 6.46) and (**b**) *m/z* 387 (1,4-NPQ + CAT, RT 11.58), respectively in the negative ionization mode (the same experimental conditions as those indicated in Figure 2). The plausible chemical structures were proposed.

Furthermore, the formation of cross-coupling products during the reaction of 1,4-NPQ with birnessite in the presence of CAT was confirmed by the results of LC-MS/MS analysis (Figure 2 and Figure 3). The oxidative transformation of CAT alone by birnessite over the 10-min reaction yielded reaction products that exhibited major total ion chromatogram (TIC) at RT 6.46 with a mass signal at m/z 217, as well as less intense signals at m/z 231, 272, 337, 339, and 459 at different RTs (Figure 2). The MS/MS analysis showed that the main signals of m/z 217 correspond to the dimeric products of CAT (M_w_ = 110.11) (Figure 3a), whereas the others may be ascribed to the various types of the reaction products generated by the birnessite-mediated oxidative coupling reaction between CAT oxidation/decomposition products (e.g., phenoxy radical, benzoquinone-like compounds, ring cleavage products, etc.) or oligomeric CAT products produced earlier. Similarly, Chang Chien et al. [25] reported that the catalytic mineralization of CAT by birnessite in air was significant, producing various aliphatic fragments by ring cleavage, and Pillar et al. [36] also obtained dimeric forms of CAT (i.e., tetrahydroxyl biphenyls at m/z 217) and reaction products of lower molecular weights ranging from m/z 73 to 101 via the heterogeneous oxidation of CAT. On the other hand, in the TIC spectrum for the reaction of 1,4-NPQ in the presence of CAT (underlined number in Figure 2), most of the signals observed for the reaction of CAT alone disappeared or significantly decreased in intensity. New major signals appeared at RT 11.58 min with MS signal of m/z 387, suggesting that 1,4-NPQ (M_w_ = 158.16) was transformed to various products via birnessite-mediated oxidative coupling in the presence of CAT. Some minor peaks having MS signals of m/z 251, 301, 331, 363, 409, and 493 also appeared at different RTs. The MS/MS spectrum of the major peak at m/z 387 showed various unidentified fragment peaks in addition to the CAT peak at m/z 109 and the main fragment peak at m/z 278, which is due to the peak of the molecular ions from which CAT was removed (i.e., M-CAT) (Figure 3b). This result indicates that the cross-coupling products of 1,4-NPQ with CAT were formed in the birnessite-CAT system (Figure S7a). The other fragment peaks could also correspond to oligomeric products from the combination of 1,4-NPQ with the decomposition products and oligomeric products of CAT in the birnessite reaction system. Similarly, Lin et al. [37] identified reaction intermediates, such as bisphenol A (M_w_ = 228.29), with low molecular weights (m/z = 110, 134, 152 etc.) produced via radical fragmentation, as well as the carbon-carbon and carbon-oxygen coupling products (m/z values including 320, 336, and 454) of the mesomeric radicals in the birnessite reaction system, using a gas chromatography (GC)-mass spectrometer (MS) analysis. These findings were further confirmed by the MS analysis after 180 min of the reaction, which revealed polymerization products with molecular sizes of up to 2000 Da (Figure S8). These results indicate that oxidative coupling reactions of the cross-coupling products of 1,4-NPQ and CAT in the presence of excess birnessite surface reaction sites proceeded with a longer incubation time. The signals at higher m/z, such as 641, 994, and 1593, may be due to oligomers of 1,4-NPQ and CAT consisting of 4 to 10 molecules or even their secondary reaction products [22] (Figure S7b). The 1,4-NPQ-phenol oxidative coupling products may present in the supernatant as a hydrophilic oligomers and/or form precipitates as the molecular weights increase and/or adsorption onto Mn oxide [15]. Similarly, Shin et al. [15] examined the fraction of insoluble reaction products formed from the 1-naphthol(1-NP) oxidative coupling reaction by Mn oxide and reported that the 1-NP contents removed by precipitation ranged from 35.7 to 48.3%.

### 3.3. 1,4-NPQ Removal by Birnessite-Catalyzed Cross-Coupling with Distinct Reaction Stages

#### 3.3.1. Effect of Birnessite Loading

**Figure 4:**
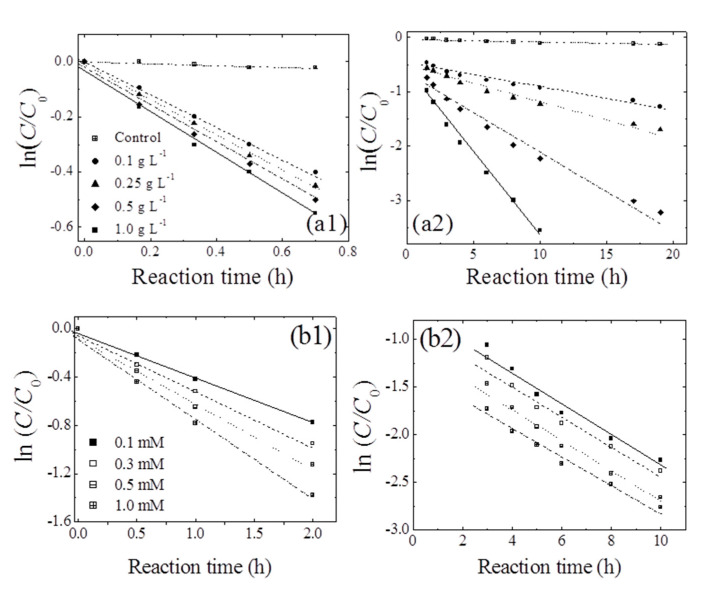
Pseudo-first-order plots for the transformation of 1,4-NPQ at different loadings of (**a**) birnessite (reaction times of (**a1**) <1 h and (**a2**) 1–24 h) and (**b**) CAT (reaction times of (**b1**) ≤ 2 h and (**b2**) 3–10 h). Experimental conditions: 0.07 mM 1,4-NPQ and (**a**) 0.3 mM catechol or (**b**) 0.5 g L^−1^ birnessite incubated in the dark at 20 °C and pH = 5.0.

Figure 4a shows the effects of birnessite loading on the removal of 1,4-NPQ in the presence of CAT (0.3 mM), which was identified as the most efficient mediator (Table 1). For all birnessite loadings, the removal efficiency sharply increased to about 35% in the first hour after mixing, slowly increasing thereafter. Notably, the extent and rate of this later-stage increase were positively correlated with birnessite loading, which was rationalized by the existence of different removal phases at different reaction times.

The data obtained for early-stage 1,4-NPQ removal were fitted to the pseudo-first-order kinetic model (r^2^ = 0.98–0.99) (Table 2). Notably, k_1_ increased slightly from 0.608 to 0.731 h^−1^ as the birnessite loading was increased from 0.1 to 1.0 g L^−1^, which demonstrates that, in the initial stages of reaction, the rate of 1,4-NPQ removal via cross-coupling reactions was not determined by the number of birnessite active sites. Therefore, the initial stage must involve the cross-coupling of 1,4-NPQ and the rapid generation of birnessite-catalyzed CAT oxidation products (e.g., o-quinones or polymerization products), as shown in Figure 2 and Figure 3 [25]. Similarly, Majcher et al. [38] and Chien et al. [25] showed that CAT is easily converted to quinone intermediates (e.g., 1,2-benzoquinone) upon its reaction with birnessite, and Park et al. [39] suggested that birnessite-catalyzed cross-coupling proceeds with high efficiency when both the phenolic mediator and target molecule form intermediates with similar structures (i.e., radical-radical or quinone-quinone). Therefore, the removal of 1,4-NPQ in the initial reaction stage is governed by the birnessite-catalyzed oxidation of CAT followed by the nucleophilic coupling of the thus-obtained oxidation products with 1,4-NPQ. The data for 1,4-NPQ removal in the later stages of the reaction was also well fitted to the pseudo-first-order model (r^2^ = 0.98–0.99). As the birnessite loading was increased from 0.1 to 1.0 g L^−1^, k_2_ increased by a factor of 7.4, and the half-life (t_1/2_) decreased from 16.9 to 2.28 h (Table 2). This increase in k_2_ with increasing birnessite loading strongly indicates that, in contrast to the initial stages, the cross-coupling of 1,4-NPQ at later reaction stages was mainly affected by birnessite-catalyzed surface reactions, i.e., surface complex formation and electron transfer reactions [30,40]. This assumption was confirmed by making a comparison of k_1_ and k_2_, which showed that the former exceeded the latter by factors of 2.4–14.8. The decrease in rate constant observed after the initial stage was attributed to the ongoing polymerization of oligomers generated by the birnessite-catalyzed cross-coupling of CAT with 1,4-NPQ, as confirmed by the detection of polymerized products after 180 min of incubation (Figure S8) and the concomitant reduction in the rate of surface complex formation. In agreement with this interpretation, Lu et al. [41] reported that the molecular weight and hydrophobicity of the products of enzyme-catalyzed phenol oxidation increased with time. Additionally, Ulrich and Stone [42] confirmed that the extent of organic complex formation at the birnessite surface decreased with the increasing hydrophobicity of the polymer product, which reduced the efficiency of birnessite-catalyzed oxidative transformations. Another plausible reason for the decrease in rate constant could be the interference with the reactive sites on the surface of birnessite by the Mn ions released from the birnessite in the reaction. Similarly, Zhang [43] observed a decrease in the rate constant in the presence of excessive manganese oxide during the removal of triclosan (5-chloro-2-(2,4-dichlorophenoxy) phenol) with an increase in the manganese ion concentration and suggested that the decrease is due to the inhibitory effect of the oxidative polymerization products of triclosan and reduced Mn ions produced during the reaction, which block the active surface sites of manganese oxide particles.

**Figure 5:**
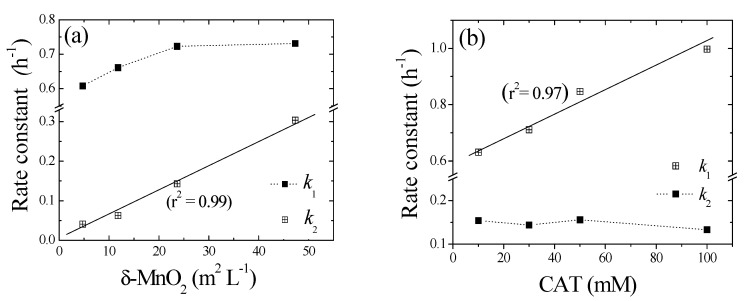
Effects of (**a**) birnessite surface area to solvent volume (m^2^ L^−1^) and (**b**) CAT concentration (mM) on pseudo-first-order rate constants (*k*_1_ and *k*_2_) for the oxidative transformation of 1,4-NPQ.

Because the cross-coupling of 1,4-NPQ occurs on the surface of the birnessite particles, the surface area is an important factor governing the reaction dynamics [40]. Therefore, we normalized the values of *k*_2_ (h^−1^) obtained from the birnessite-loading experiments using the surface area of birnessite (44.37 m^2^ g^−1^) (e.g., birnessite loading (g L^−1^) × surface area (m^2^ g^−1^)) to obtain surface-area-normalized rate constants (*K*_surf_, L m^−2^ h^−1^), as shown in Figure 5a. The *k*_2_ values exhibited a linear (*r*^2^ = 0.99) dependence on the “birnessite surface area concentration (m^2^ L^−1^)” and *K*_surf_ was determined from the slope of the corresponding plot (0.07 mM 1,4-NPQ, 0.3 mM CAT, 0.1–1.0 g L^−1^ *δ*-MnO_2_, pH = 5). The obtained *K*_surf*,*_ value (6.36 × 10^−4^ L m^−2^ h^−1^) was about 90 times lower than the value previously reported for 1-naphthol (5.6 × 10^−2^ L m^−2^ h^−1^), which has a similar structure to 1,4-NPQ [15]. The results indicate that the birnessite-catalyzed cross-coupling was much slower than the well-known birnessite-mediated oxidative coupling of phenolic compounds. Furthermore, the derived *K*_surf_ value was shown to be independent of birnessite dosage and, thus, provides more generalized information for the quantitative assessment of birnessite reactivity.

#### 3.3.2. Effect of CAT Loading and Reaction Sequence

Figure 4b shows the temporal profiles of 1,4-NPQ removal at various CAT concentrations, revealing that the removal efficiency increased with increasing CAT concentration in the initial stages and was independent of CAT concentration in the later stages. Interestingly, k_1_ increased from 0.631 to 0.997 h^−1^ as the CAT concentration increased from 0.1 to 1.0 mM (Table 2) and stabilized at 0.133–0.154 h^−1^ in the later stages. These results indicate that, early on, the birnessite-catalyzed removal of 1,4-NPQ was primarily governed by the formation of oxidative CAT coupling products (e.g., o-quinones and phenoxy radicals), whereas the CAT concentration did not significantly affect the rate of 1,4-NPQ removal in subsequent reactions because CAT was rapidly consumed in the fast reaction with birnessite in the initial reaction stage (Figure S9b). This finding is consistent with the results of the birnessite loading experiments (Figure 4a) and confirms that the initial removal of 1,4-NPQ was mainly dominated by the cross-coupling reaction of 1,4-NPQ and the product(s) of CAT oxidation rather than the surface-controlled reaction influenced by the amount of added birnessite [32,44].

The above conclusions were verified by examining the effects of changing the reaction sequence. Table 3 shows the kinetic data for 1,4-NPQ removal acquired in the B1 and B2 experiments with birnessite loadings of 0.5 and 1.0 g L^−1^, which demonstrate that the B1 k_1_ values exceeded those of B2. This behavior can be ascribed to the fact that, in the B2 experiments, the efficiency of birnessite-catalyzed CAT oxidation products as mediators for 1,4-NPQ cross-coupling was lower than that of CAT itself because CAT was gradually converted to polymeric products. This interpretation is supported by the data in Figure S6b), which show that CAT had almost been completely removed (>99.9%) after a 20-min exposure to birnessite, accompanied by the appearance of polymeric product peaks. Ulrich and Stone [42] and Lu et al. [41] have also reported that the organic matter sorption ability and electron-transfer efficiency of the birnessite surface decrease with increasing extent of the birnessite-catalyzed oxidative polymerization of phenolic compounds, which reduces the reaction efficiency. On the other hand, the ratio of k_1_ obtained for the two different batches (i.e., k_1(B1)_/k_1(B2)_) decreased from 2.2 to 1.7 as the birnessite loading increased from 0.5 to 1.0 g L^−1^, whereas the corresponding k values observed for the B1 and B2 experiments at later stages (2–24 h) were similar, in good agreement with those of the birnessite and CAT loading experiments. Thus, the initial stage of 1,4-NPQ removal was dominated by the cross-coupling of 1,4-NPQ and the products of CAT oxidation, whereas the later stages were dominated by birnessite-catalyzed surface reactions of the initially produced cross-coupling products and the products of CAT polymerization.

## 4. Conclusions

We studied the removal of 1,4-NPQ by birnessite-catalyzed oxidation in the presence of phenolic mediators and determined the effect of the phenolic mediator structure on the efficiency of 1,4-NPQ removal. We also characterized the corresponding reaction pathway. The results showed that the high removal efficiencies of 1,4-NPQ were obtained for diphenols and phenols bearing electron-donating substituents (e.g., -OCH_3_ and -OH), and the removal reaction was more effective for o- and p- than m-substituents. This was confirmed by the high correlation between the k_1_ rate constant for 1,4-NPQ removal and the Hammett constants of the phenolic mediators showing a negative sign of the susceptibility factor (i.e., *ρ* = −0.61). The analysis of the effects of the birnessite and CAT loadings on the efficiency and kinetics of 1,4-NPQ removal confirmed that the cross-coupling of 1,4-NPQ proceeds via two different reaction stages. In the early reaction stage, CAT acts as the major limiting factor for the cross-coupling reaction of 1,4-NPQ, and, in later-stage reactions, birnessite acts as the limiting factor. Specifically, the faster initial removal stage is dominated by the cross-coupling reaction of 1,4-NPQ and the rapidly formed oxidized phenolic mediator (e.g., phenoxy radical), whereas the later stages of the reaction proceed more slowly with 1,4-NPQ polymerized reaction products dominating the surface reactions that are directly catalyzed by birnessite, as confirmed by the analysis of mixing order experiments.

This study provides new information on the mechanism and kinetics of the birnessite-catalyzed cross-coupling of a representative quinoid PAH compound in the presence of phenolic mediators, and the use of the same principal in engineered restoration processes is a promising technology for remediating soils and water contaminated with PAH degradation products (e.g., oxygenated PAHs).

## Figures and Tables

**Table 1:** Efficiency of 1,4-naphthoquinone (NPQ) oxidation in various birnessite-phenolic mediator systems.

Phenolic Mediator		RT (min) ^a^	Efficiency (%) ^b^	Kinetic Parameters	
				k_1_^c^ (× 10^−1^, h^−1^)	k_2_^d^ (× 10^−1^, h^−1^)
None (control) ^e^		-	1.7 ± 0.1	<0.001	<0.001
*δ*-MnO_2_ only ^f^		-	9.8 ± 0.8 (3.0)	0.28 (0.81)	0.03 (0.89)
Diphenol	Catechol (CAT)	1.83	100 ± 0.3 (82.3)	7.33 (0.98)	4.83 (0.98)
	Hydroquinone (HQ)	2.18	100 ± 0.4 (65.0)	6.11 (0.98)	4.08 (0.98)
	Resorcinol (RES)	1.75	100 ± 0.8 (46.7)	3.80 (0.98)	3.66 (0.98)
Methoxy phenol	4-Methoxyphenol (4-MeP)	2.25	99.8 ± 0.4 (47.6)	4.00 (0.98)	4.03 (0.99)
	2,6-Dimethoxyphenol (2,6-DiMeP)	2.36	99.9 ± 0.5 (71.6)	6.57 (0.98)	6.16 (0.98)
Acidic phenol	Syringic acid (SyA)	2.61	77.6 ± 1.2 (15.9)	0.71 (0.96)	0.64 (1.00)
	Vanillic acid (VA)	2.38	79.5 ± 0.5 (22.6)	1.18 (0.97)	1.11 (1.00)
	3,4-Dihydroxybenzoic acid (3,4-DiHBA)	2.33	94.9 ± 0.7 (38.7)	2.44 (0.97)	1.51 (1.00)
	Ferulic acid (FA)	2.46	93.6 ± 0.8 (15.5)	0.75 (0.98)	0.59 (1.00)
Aldehyde phenol	Syringic aldehyde (SyAl)	2.61	98.1 ± 1.2 (27.3)	1.56 (0.98)	1.60 (1.00)
	Vanillin (Val)	2.25	87.8 ± 1.1 (16.4)	0.79 (0.96)	0.80 (1.00)

^a^ Retention time from an HPLC chromatogram. ^b^ Experimental conditions: 0.07 mM 1,4-NPQ, 1.0 g L^−1^*δ*-MnO_2_, 0.3 mM phenolic mediator, incubation in the dark for 24 h at 20 °C, and pH = 5 (values in parentheses denote the percent initial removal efficiency measured at 1.5 h for diphenols and methoxy phenols and 2 h for acidic and aldehyde phenols). The reaction time was chosen based on the coefficients of determination (*r*^2^ ≥ 0.98) from the pseudo-first-order kinetic model fitting. ^c^ Initial rate constant for 1,4-NPQ removal for reaction time at 1.5 (CAT to 2,6-DiMeP) and 2 h (SyA to Val) (values in parentheses denote the coefficient of determination). ^d^ Rate constant for 1,4-NPQ removal for a reaction time of 1.5–24 h (CAT to 2,6-DiMeP) and 2–24 h (SyA to Val) (values in parentheses denote the coefficient of determination). ^e^ Without *δ*-MnO_2_ and phenolic mediator. ^f^ Without phenolic mediator.

**Table 2:** Kinetic data for birnessite-catalyzed removal of 1,4-NPQ in the presence of catechol (CAT).

	**Birnessite loading (g L^−1^) ^a^**			
	**0.1**	**0.25**	**0.5**	**1.0**
*k*_1_ (h^−1^) ^b^	00.608	00.651	0.723	0.731
*r* ^2^	00.990	00.990	0.990	0.990
*t*_1/2_ (h)	01.140	01.060	0.960	0.950
*k*_2_ (h^−1^) ^c^	00.041	00.063	0.143	0.304
*r* ^2^	00.990	00.990	0.990	0.990
*t*_1/2_ (h)	16.900	11.000	4.850	2.280
*K*_surf_ (L m^−2^ h^−1^) ^d^	6.36 × 10^−4^			
	**CAT loading (mM) ^e^**			
	**0.1**	**0.3**	**0.5**	**1.0**
*k*_1_ (h^−1^) ^b^	00.631	00.711	0.847	0.997
*r* ^2^	00.990	00.980	0.990	0.980
*t*_1/2_ (h)	01.100	00.990	0.820	0.700
*K*_cat_ (L mM^−1^ h^−1^) ^f^	0.383			
*k*_2_ (h^−1^) ^c^	00.154	00.144	0.156	0.133
*r* ^2^	00.960	00.960	0.980	0.980
*t*_1/2_ (h)	04.500	04.810	4.440	5.210

^a^ Experimental conditions: 0.07 mM 1,4-NPQ, 0.3 mM CAT, variable birnessite loading, incubation in the dark at 20 °C and pH = 5. ^b^ Initial rate constant in the reaction time < 1.0 h (for birnessite loading) and < 2 h (for CAT loading) based on the coefficient of determination (r^2^ ≥ 0.98). ^c^ Rate constant in the reaction time from 1.0 to 24 h (for birnessite loading) and 2.0 to 24 h (for CAT loading). ^d^ Obtained after normalizing birnessite loading (g L^−1^) to birnessite surface area (m^2^ L^−1^) using the birnessite surface area of 47.3 m^2^ g^−1^. ^e^ Experimental conditions: 0.07 mM 1,4-NPQ, 0.5 g L^−1^ *δ*-MnO_2_, variable CAT loading, incubation in the dark at 20 °C and pH = 5. ^f^ Normalized to CAT concentration (mM).

**Table 3:** Kinetic constants for different reaction sequences of the cross-coupling of 1,4-NPQ in the presence of catechol.

Birnessite Loading (g L^−1^) ^a^	Reaction Sequence	*k*_1_ (h^−1^) ^b^	*r* ^2^	*t*_1/2_ (h)	*k_2_* (h^−1^) ^c^	*r* ^2^	*t*_1/2_ (h)
0.5	Batch 1 ^d^	0.847	0.97	0.82	0.156	0.94	4.44
	Batch 2 ^e^	0.389	0.96	1.78	0.159	0.95	4.35
1.0	Batch 1 ^d^	0.935	0.96	0.74	0.298	0.94	2.33
	Batch 2 ^e^	0.555	0.97	1.25	0.274	0.95	2.53

^a^ Experimental conditions: 0.07 mM 1,4-NPQ, 0.5 mM CAT, variable birnessite loading, incubation in the dark at 20 °C and pH = 5. ^b^ Initial rate constant for reaction time (≤1.5 h, *r*^2^ ≥ 0.96). ^c^ Rate constant in the reaction time from 1.5 to 24 h. ^d^ Simultaneous mixing of birnessite, 1,4-NPQ, and CAT. ^e^ Mixing of birnessite and CAT followed by the addition of 1,4-NPQ after 30 min.

## Supplementary Materials

The following are available online at https://www.mdpi.com/1660-4601/17/13/4853/s1, Figure S1: Schematic illustration of the experimental design, Figure S2: X-ray diffraction patterns of the synthesized birnessite, Figure S3: The SEM analysis result of the synthesized birnessite, Figure S4: Time profiles of 1,4-NPQ removal by birnessite-catalyzed oxidation in the presence of different phenolic mediators. Experimental conditions: 0.07 mM 1,4-NPQ, 0.3 mM phenolic mediator, 1.0 g L^−1^ *δ*-MnO_2_, 20 °C, and pH = 5 in the dark, Figure S5: Correlation between the initial rate constant (*k*_1_) and rate constant (*k*_2_) of later reaction stages for 1,4-NPQ removal in the presence of CAT (same experimental condition as Table 1), Figure S6: HPLC chromatograms of (a) 1,4-NPQ and products of its birnessite-catalyzed oxidation in the presence of catechol and (b) catechol and products of its birnessite-catalyzed oxidation recorded at different times. Experimental conditions: 0.07 mM 1,4-NPQ, 0.3 mM catechol, and 1.0 g L^−1^ *δ*-MnO_2_ incubated in the dark at 20 °C and pH = 5, Figure S7: Proposed removal pathways of 1,4-NPQ by birnessite in the presence of the phenolic mediator, Figure S8: MS spectrum of the reaction products for a 180-min incubation time (Same experimental conditions as listed in Figure 2 and a retention time of 8.0 min, Figure S9: Disappearance of 1,4-NPQ in aqueous suspension at different (a) birnessite and (b) catechol loadings. Experimental conditions: 0.07 mM 1,4-NPQ and (a) 0.3 mM catechol or (b) 0.5 g L^−1^ birnessite incubated in the dark at 20 °C and pH = 5.0, Table S1: Physicochemical properties of 1,4-NPQ and phenolic mediators used in this study, Table S2: Amounts of removed 1,4-NPQ and CAT compounds and dissolved and adsorbed Mn in the birnessite reaction system.
