# Supplementary material for: Removal of 1,4-Naphthoquinone by Birnessite-Catalyzed Oxidation: Effect of Phenolic Mediators and the Reaction Pathway
Source: Int J Environ Res Public Health. 2020 Jul 6;17(13):4853. doi: 10.3390/ijerph17134853 (PMC7370181; doi:10.3390/ijerph17134853)
Supplement: Supplementary file 1 [file ijerph-17-04853-s001.pdf]

Supplementary materials

Removal of 1,4-Naphthoquinone by Birnessite-Catalyzed Oxidation: Effect of Phenolic Mediators and Reaction Pathway

Han-Saem Lee [1], Jin Hur [2], Doo-Hee Lee[3], Mark A. Schlautman[4], and Hyun-Sang Shin [1,*]

[1] Department of Environment Energy Engineering, Seoul National University of Science & Technology; hansun213@seoultech.ac.kr
[2] Department of Environment & Energy, Sejong University; jinhur@sejong.edu
[3] Mass Spectrometer Laboratory, National Instrumentation Center for Environmental Management; dohe80@snu.ac.kr
[4] Department of Environmental Engineering and Earth Science, Clemson University; mschlau@clemson.edu
* Correspondence: hyuns@seoultech.ac.kr; Tel.: +82-2-970-6625

Table S1 Physicochemical properties of 1,4-NPQ and phenolic mediators used in this study.

Compound		Formula/M_w (g mol^{-1})	Mediator substituent[a]	S_w[b] (mg L^{-1}, × 10^4)	log K_{ow}[c] (L kg^{-1})	$\Sigma\sigma_{o,\,m,\,p}$[d]
1,4-Naphthoquinone (1,4-NPQ)		$C_{10}H_6O_2$ 158.16	-	000.07	1.71	
Phenolic mediator	Catechol (CAT)	$C_6H_6O_2$ 110.11	2(OH)	046.10	0.88	-0.20[1)]
	Hydroquinone (HQ)	$C_6H_6O_2$ 110.11	4(OH)	007.20	0.59	-0.37[1)]
	Resorcinol (RES)	$C_6H_6O_2$ 110.11	3(OH)	110.00	0.80	0.12[1)]
	4-Methoxyphenol (4-MeP)	$C_7H_8O_2$ 124.13	4(OCH_3)	004.00	1.58	-0.27[1)]
	2,6-Dimethoxyphenol (2,6-DiMeP)	$C_8H_{10}O_3$ 154.16	2,6(OCH_3)	001.72	1.15	-0.42[1)]
	Syringic acid (SyA)	$C_9H_{10}O_5$ 198.17	2,6(OCH_3), 4(COOH)	000.58	1.04	0.68[3)]
	Vanillic acid (VA)	$C_8H_8O_4$ 168.15	2(OCH_3), 4(COOH)	000.15	1.43	0.49[1)]
	3,4-Dihydroxybenzoic acid (3,4-DiHBA)	$C_7H_6O_4$ 154.12	2(OH), 4(COOH)	001.82	0.86	0.35[2)]
	Ferulic acid (FA)	$C_{10}H_{10}O_4$ 194.18	2(OCH_3), 4(COOH-CH=CH-)	000.60	1.42	0.03[2)]
	Syringic aldehyde (SyAl)	$C_9H_{10}O_4$ 198.17	2,6(OCH_3), 4(CHO)	000.53	1.88	0.55[3)]
	Vanillin (Val)	$C_8H_8O_3$ 152.15	2(OCH_3), 4(CHO)	001.10	1.12	0.43[2)]

[a]Number in front of substituent refers to position on phenol ring.

[b]S_w: Water solubility at 20–25 °C.

[c]K_{ow}: 1-Octanol/water partition coefficient.[d] Hammett constants from reference [1)](Díaz et al. 1998), [2)](Takahata and Chong 2005), and [3)](Gan et al. 2019).

Table S2 Amounts of removed 1,4-NPQ and CAT compounds and dissolved and adsorbed Mn in the birnessite reaction system.[a]

Birnessite reaction system	Removed compounds (μmol)	Reduced Mn (μmol)		Ratio of oxidation/ reduction[b]
		Dissolved	Adsorbed	
Birnessite only	N.A.[c]	N.D.[d]	N.D.[d]	N.A.[c]
1,4-NPQ	6.7 (≈ 9.8%)	0.44	1.46	2.01
1,4-NPQ + CAT	370 (> 99.5%)	67.2	88.3	2.38
CAT	300 (> 99.5%)	47.1	50.9	3.05

[a]Experimental conditions: 0.07 mM 1,4-NPQ, 1.0 g L^{-1} δ-MnO_2, 0.3 mM phenolic mediator, incubation in the dark for 24 h at 20 °C, and pH 5.

[b]Ratio of the amount of degraded phenol or 1,4-NPQ compounds to that of the sum of dissolved Mn and adsorbed Mn.

[c]Not applicable.

[d]Not detectable.

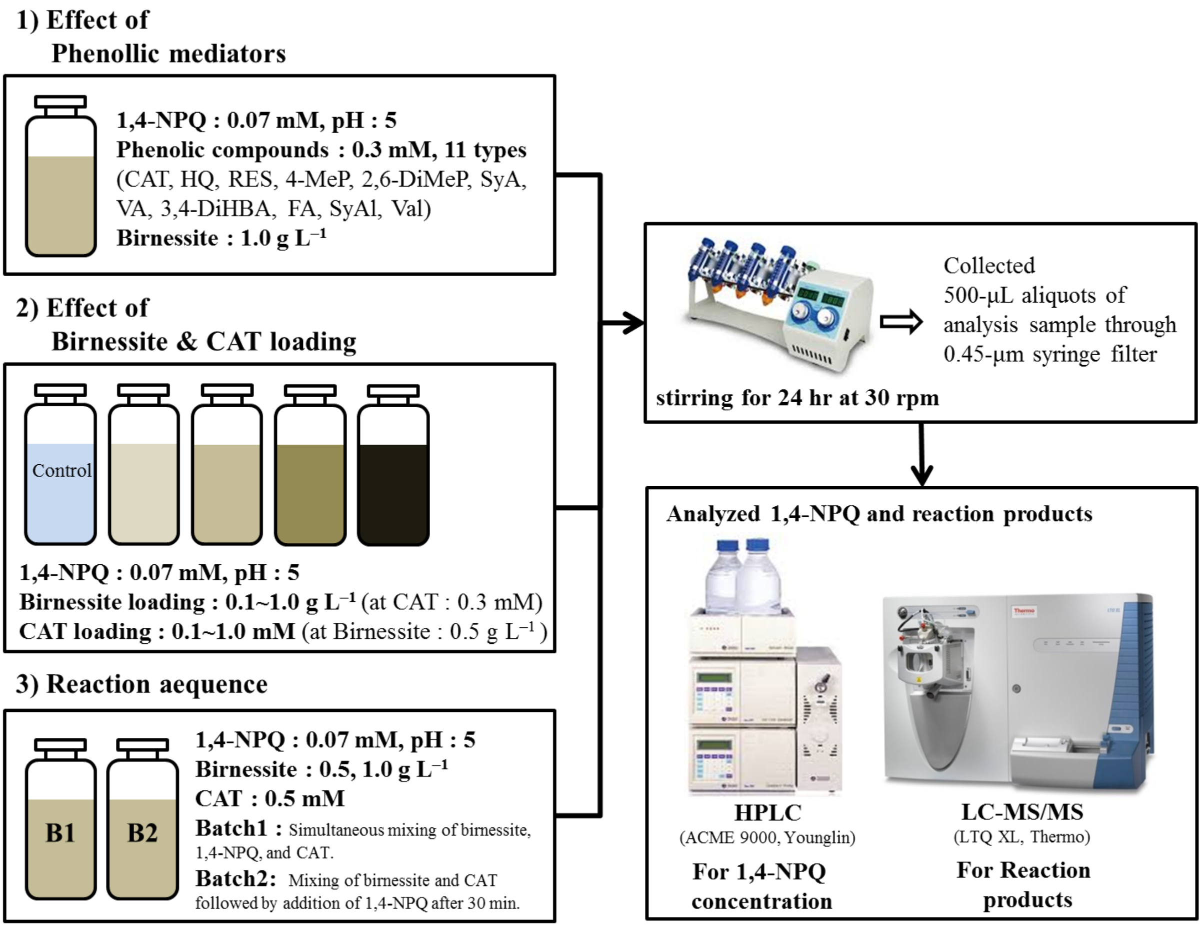

Fig. S1 Schematic illustration of the experimental design

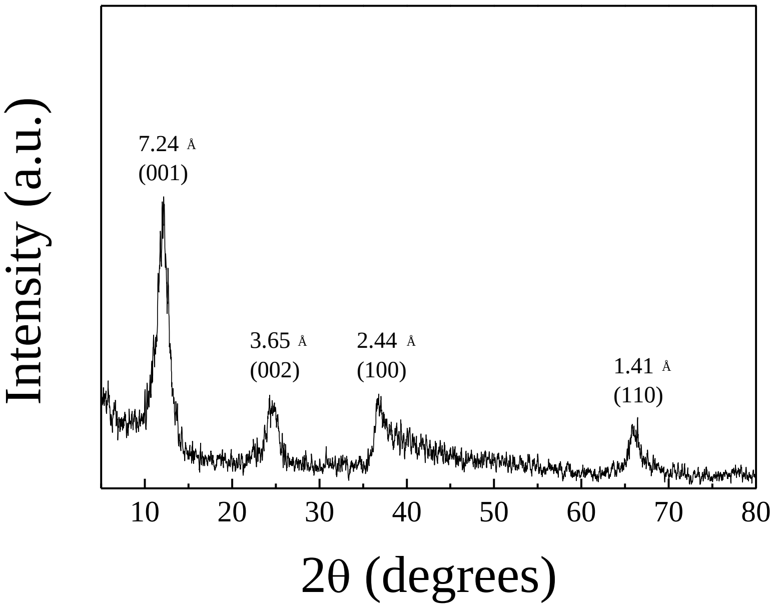

Fig. S2 X-ray diffraction patterns of the synthesized birnessite.

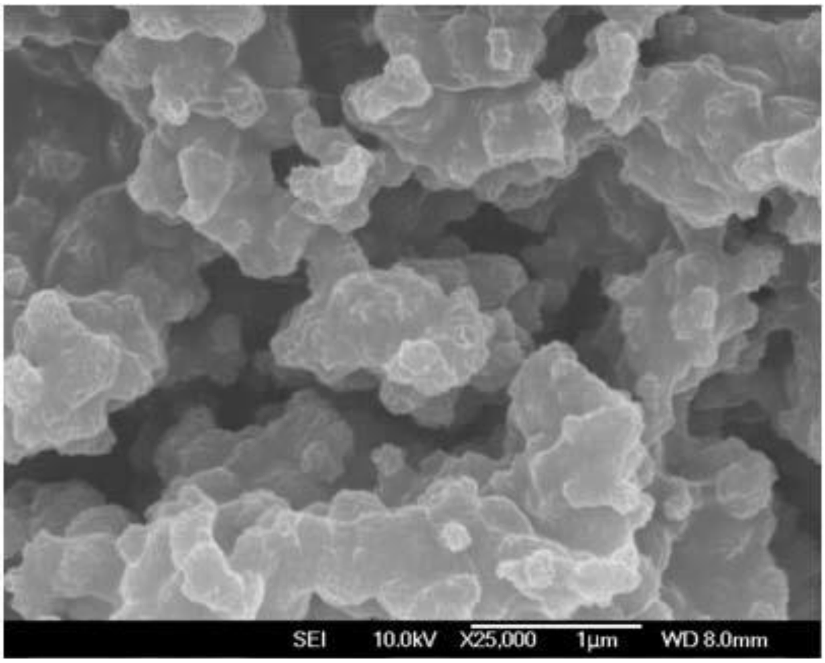

Fig. S3 The SEM analysis result of the synthesized birnessite.

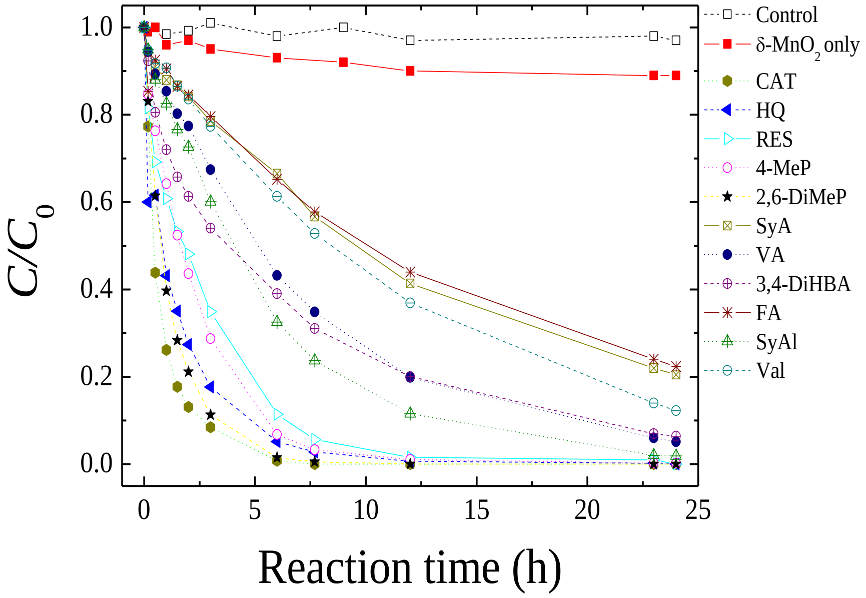

Fig. S4 Time profiles of 1,4-NPQ removal by birnessite-catalyzed oxidation in the presence of different phenolic mediators. Experimental conditions: 0.07 mM 1,4-NPQ, 0.3 mM phenolic mediator, 1.0 g L^{-1} δ-MnO_2, 20 °C, and pH 5 in the dark.

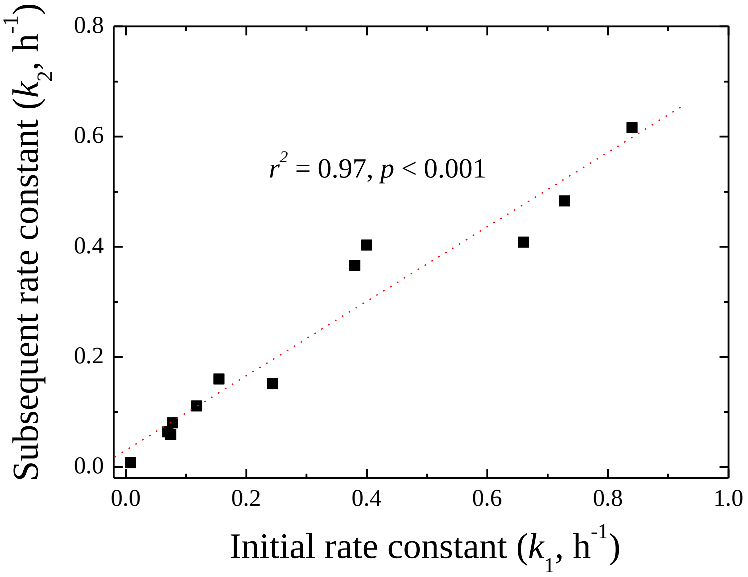

Fig. S5 Correlation between the initial rate constant (k_1) and rate constant (k_2) of later reaction stages for 1,4-NPQ removal in the presence of CAT (same experimental condition as **Table 1**).

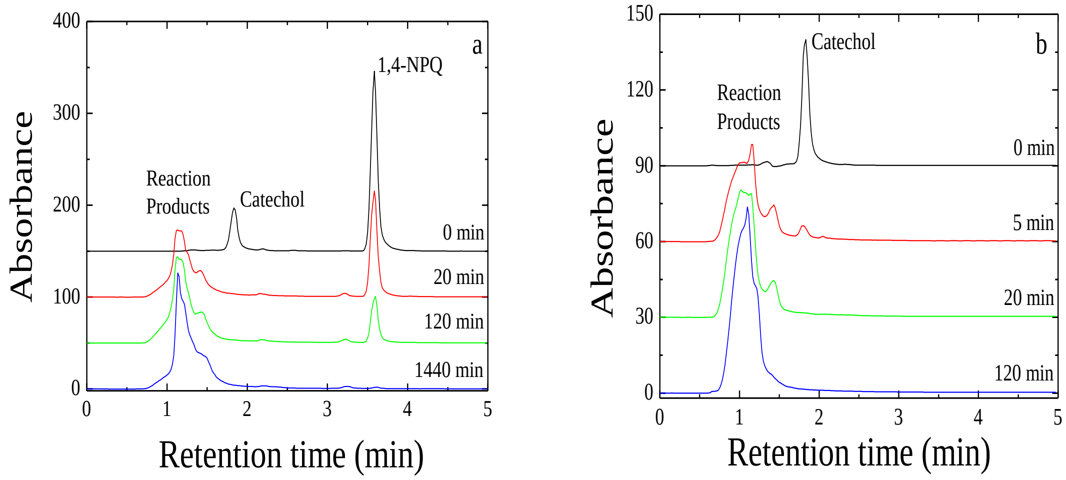

Fig. S6 HPLC chromatograms of (a) 1,4-NPQ and products of its birnessite-catalyzed oxidation in the presence of catechol and (b) catechol and products of its birnessite-catalyzed oxidation recorded at different times. Experimental conditions: 0.07 mM 1,4-NPQ, 0.3 mM catechol, and 1.0 g L^{-1} δ-MnO_2 incubated in the dark at 20 °C and pH 5.

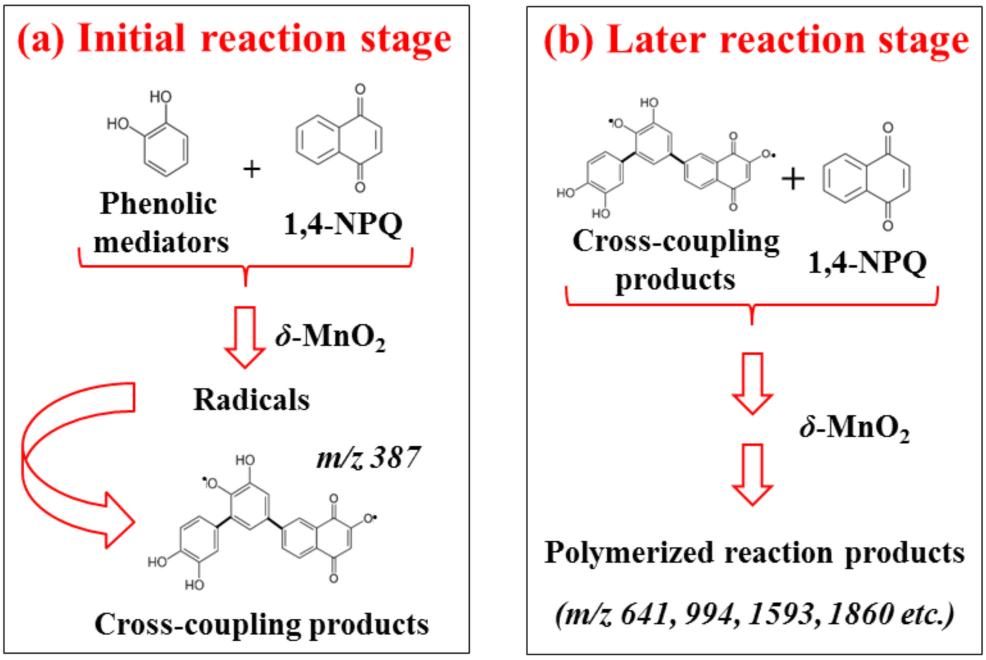

Fig. S7 Proposed removal pathways of 1,4-NPQ by birnessite in the presence of the phenolic mediator.

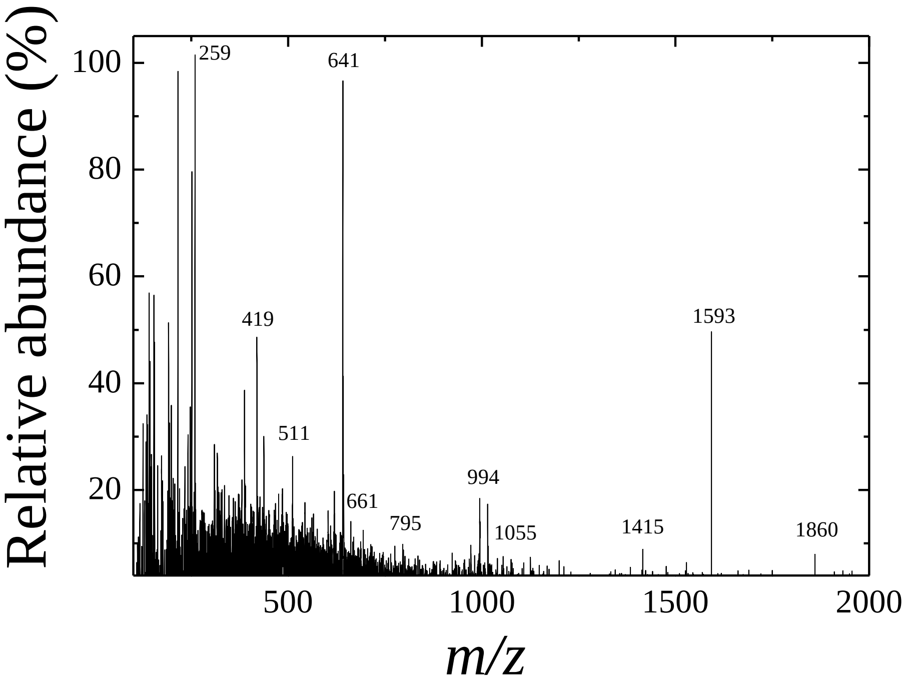

Fig. S8 MS spectrum of the reaction products for a 180-min incubation time (Same experimental conditions as listed in **Fig. 2** and a retention time of 8.0 min).

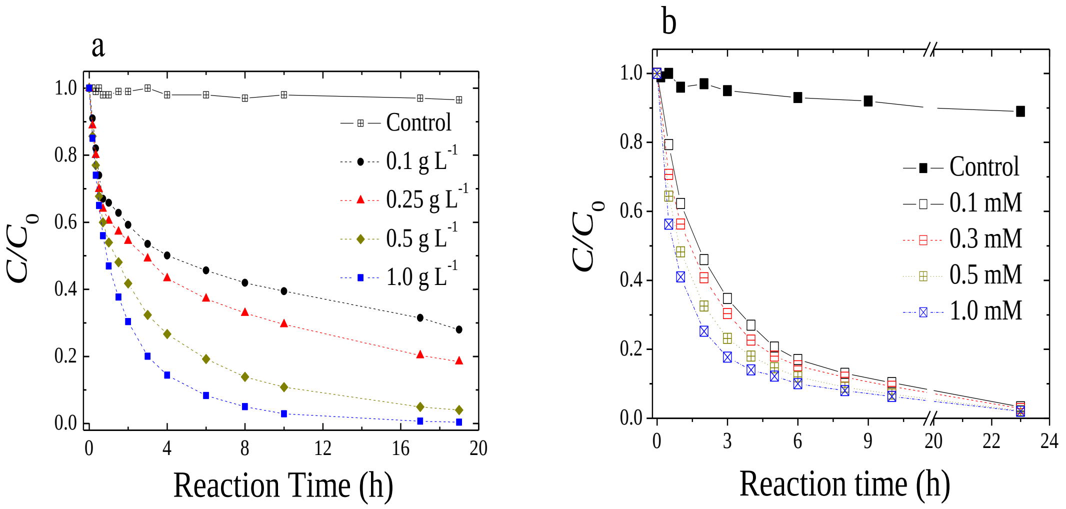

Fig. S9 Disappearance of 1,4-NPQ in aqueous suspension at different (a) birnessite and (b) catechol loadings. Experimental conditions: 0.07 mM 1,4-NPQ and (a) 0.3 mM catechol or (b) 0.5 g L^{-1} birnessite incubated in the dark at 20 °C and pH 5.0.

References

Díaz, A.N., Sánchez, F.G., González Garcia, J.A. Phenol derivatives as enhancers and inhibitors of luminol-H_2O_2-horseradish peroxidase chemiluminescence. *J. Biolumin. Chemilumin.* **1998**, *13*, 75–84.

Gan, W., Ge, Y., Zhu, H., et al. ClO_2 pre-oxidation changes the yields and formation pathways of chloroform and chloral hydrate from phenolic precursors during chlorination. *Water Res.* **2019**, *148*, 250–260.

Takahata, Y., Chong, D.P. Estimation of Hammett sigma constants of substituted benzenes through accurate density-functional calculation of core-electron binding energy shifts. *Int. J. Quantum. Chem.* **2005**, *103*, 509–515.
